# Human bocavirus (HBoV) in children with respiratory tract infection by enzyme linked immunosorbent assay (ELISA) and qualitative polymerase chain reaction (PCR)

**DOI:** 10.1186/1743-422X-8-239

**Published:** 2011-05-19

**Authors:** Mona Z Zaghloul

**Affiliations:** 1 Department of Clinical Pathology, Ain Shams University Hospitals, Cairo, Egypt

## Abstract

**Background:**

Human bocavirus (HBoV) is a recently discovered parvovirus associated with mild to severe lower respiratory tract infections in children, the aim of the work was determination of human bocavirus in nasopharyngeal aspirate (NPA) of infants by qualitative PCR and determination of acute human bocavirus infection by estimation of immunoglobulin M (IgM) antibodies in serum by enzyme linked immunosorbent assay.

**Results:**

Twenty two (22%) out of the 100 NPA specimens of the patients with respiratory manifestations were positive for HBoV by qualitative PCR, while ELISA revealed positive HBoV IgM antibodies in 18 (18%) patients who were also positive by PCR. Non of the controls were positive by both techniques. The correlation study between ELISA and PCR revealed high significant association, (p < 0.001, X^2 ^= 36 and agreement = 96%). Also PCR detected 4 (18.1%) NPA samples as HBoV positive cases among the patients that were not identified by ELISA. This could be due to high sensitivity and efficacy of PCR. ELISA being less sensitive than RT-PCR, sensitivity was (81.8% vs 100%), the efficacy was 97.7% in ELISA versus 99.7% for RT-PCR.

**Conclusion:**

HBoV infections could be diagnosed in NPA of children by conventional PCR as a rapid and sensitive technique. While ELISA was a reliable serologic analysis for diagnosis of acute HBoV infection by estimation IgM antibodies in serum.

## Background

In 2005, Allander et al., [1] reported the discovery of a previously undescribed human parvovirus in respiratory secretions from children with respiratory tract disease in Sweden. Phylogenetic analysis showed that this virus belonged to the genus *Bocavirus *(subfamily, *Parvovirinae*; family, *Parvoviridae*) and was most closely related to bovine parvovirus (BP) and minute virus of canines (MVC). The virus was thus named "human bocavirus" (HBoV).

Human bocaviruses (HBoV) contain 3 open reading frames (ORFs) encoding a nonstructural protein (NS1, NP1) and two capsid proteins VP1 and VP2, respectively [2]. The genomic organization of HBoV closely resembles that of bovine parvovirus type 1 [1].

HBoV has been reported in respiratory samples from children with acute respiratory tract disease in various parts of the world (including Australia, North America, Europe, Asia, and Africa), suggesting that the virus is circulating worldwide [3]. Pneumonia, acute bronchitis, bronchiolitis, are the main manifestations of HBoV infection [4].

HBoV seems to be a new member of the community-acquired respiratory viruses such as respiratory syncytial virus, adenovirus, influenza virus, parainfluenza virus, and rhinovirus, which cause common respiratory tract infections in the community [5]. Because of its very high copy numbers in respiratory tract secretions, aerosol and contact transmission are likely effective, as they are for other respiratory viruses [6].

HBoV has been detected also in nasopharyngeal, serum, fecal and urine samples obtained mainly from young children around the age of 2 years predominantly during the winter season [1]. HBoV was detected in two pediatric patients after organ transplantation [7], in human immunodeficiency virus-infected pediatric patients [8], and immunosuppressed adult patients [9].

Diagnosis of HBoV infection is based on the PCR amplification of viral genome fragments present in human respiratory, serum, stool and urine samples. A great number of different PCR techniques employing varying sets of primers specific for the viral genes NP1[10], NS1 [11], and VP1, VP2 [12,13] have been described. In addition to the detection of viral genomes by PCR, recent reports describe the detection of HBoV-specific IgG and IgM-antibodies against HBoV VP2 in serum samples using western blot [14] or immunofluorescence assays [2,15]. Furthermore, a standardized ELISA for the quantitative determination of HBoV-specific antibodies has been established by [16]. The aim of the work was determination of HBoV in respiratory specimens (NPA) of infants by qualitative PCR and determination of acute HBoV infection by estimation of IgM antibodies in serum by ELISA.

## Results

### Microbiological results

Cultures of nasopharyngeal aspirates from 100 patients were positive in 67% of patients. Cultures yielded growth of one or more pathogen among upper respiratory tract normal flora. Gram positive cocci were isolated from 57/90 (63.3%) of specimens including: *Streptococcus pneumoniae *25/90 (27.7%)*, Staphylococcus aureus *32/90 (35.5%). Gram negative bacilli were detected in 33/90(36.6%) of specimens including, *Klebsiella pneumoniae *15/90 (16.6%), *Escherichia coli *10/90 (11.1%), *Pseudomonas aeruginosa *8/90 (8.8%), while Cultures of the control group showed growth of upper respiratory tract normal flora (table 1).

**Table 1:** The isolated bacteria in NPA specimens of patients with respiratory tract infection

Organism	Number (%)	Organism spp.	IsolateNumber (%)
**Gram positive cocci**		*Streptococcus pneumoniae*	25/90 (27.7%)
		
	57/90(63.3%)	*Staphylococcus aureus*	32/90 (35.5%)
		
**Gram negative bacilli**		*Klebsiella pneumoniae*	15/90 (16.6%)
		
	33/90(36.6%)	*E. coli*	10/90 (11.1%)
		
		*Pseudomonas aeruginosa*	8/90 (8.8%)

**Normal flora**	33 (33%)		

**Total**	100 (100%)		

### Qualitative PCR results

Twenty two (22%) out of the 100 NPA specimens of the patients were positive for HBoV and 78 (78%) specimens were negative by qualitative PCR. While all the 50 NPA of the control group were negative for HBoV. Also there was a high significant difference between the patient and the control groups since p < 0.001 (table 2).

**Table 2:** Comparison between patients and controls by PCR

Groups	PCR +ve	PCR -ve	(%)
**Patients** **n = 100**	**22**	**78**	**22%**

**Controls** **n = 50**	**0**	**50**	**0**

P < 0.001 (highly significant)

### ELISA results

Eighteen (18%) out of the 100 serum samples of the patients with respiratory tract manifestations were positive for HBoV IgM antibodies by ELISA such patients are also positive by PCR, and 82 (82%) specimens were HBoV negative by ELISA. While all 50 serum samples of the controls were negative (table 3). Serum concentration of IgM antibodies against HBoV were detected significantly in patients (0.28 ± 0.06 vs 0.07 ± 0.01 controls), p < 0.001. Table 4 showed the number of positive cases by the different diagnostic methods. Table 5 showed that there was a highly significant association between the PCR and ELISA techniques.

**Table 3:** Comparison between patients and controls by ELISA.

Groups	ELISA+ve	ELISA -ve	(%)
**Patients** **n = 100**	**18**	**82**	**18%**

**Controls** ** n = 50**	**0**	**50**	**0**

P < 0.001 (highly significant)

**Table 4:** Number of positive cases by the different diagnostic methods.

Method	No. of HBoV (+) cases		No. of HBoV (-) cases		Total
	N	(%)	N	(%)	
**PCR**	22	22	78	78	100

**ELISA**	18	18	82	82	100

**Table 5:** Correlation between PCR and ELISA.

		ELISA (-)	ELISA (+)	Total
**PCR (-)**	**Number**	78	0	78
	
	**%/total**	95.1	0	78
	
**PCR (+)**	**Number**	4	18	22
	
	**%/total**	4.9	100	22

**Total**		82	18	100

X^2 ^= 36, P < 0.001, Agreement = 96%

### Diagnostic validity tests

Diagnostic validity test including sensitivity, specificity, predictive values and efficacy of both HBoV ELISA and PCR were calculated (table 6 and 7) considering PCR as a reference method. In our work we found ELISA to be less sensitive than PCR, it was (81.8% vs 100%), but the specificity of ELISA is higher than PCR, it was (100% vs 78%) (table 6 and 7).

**Table 6:** Diagnostic validity test of ELISA.

True positive	False positive		True negative	False negative
18	0		78	4

**Sensitivity%**	**Specificity%**	**Positive predictive%**	**Negative predictive %**	**Efficacy%**

81.8%	100%	100%	95.1%	97.7%

**Table 7:** Diagnostic validity test of PCR.

True positive	False positive		True negative	False negative
22	0		78	0

**Sensitivity%**	**Specificity%**	**Positive predictive%**	**Negative predictive %**	**Efficacy%**

100%	78%	100%	100%	99.7%

## Discussion

In recent years, several novel viruses have been discovered in patients with respiratory infections using molecular biology methods. These novel viruses include the human metapneumovirus and several coronaviruses (SARS, NL63, and HKU1) [17,18] the latest addition to this list was the human bocavirus (HBoV).

Attempts to culture this virus on conventional cell lines had failed but, Dijkman et al., [19] had investigated whether the virus can replicate on pseudostratified human airway epithelium. The cells were inoculated with human bocavirus-positive nasopharyngeal washes from children, and virus replication was monitored by measuring apical release of the virus via real-time PCR.

Electron Microscopy supported the ultrastructure analysis of the HBoV virus like particles (VLP) using a transmission electron microscope equipped with digital camera [20].

In our work 22 (22%) out of the 100 NPA specimens of the patients were positive for HBoV, There was a high significant difference between the patient and the control groups (p < 0.001). PCR detected four HBoV positive cases that were negative by ELISA, this could be explained by the high sensitivity of PCR over ELISA since it was (100% vs 81.1%) respectively. Weissbrich et al., [21] had found HBoV DNA in (10.3%) of NPA samples obtained from 786 hospitalized infants and children with febrile respiratory tract diseases during the years 2002 to 2005 in the region of northern Bavaria in Germany. While Neske et al., [22] had studied 834 nasopharyngeal aspirates (NPA), 10 serum samples, and 31 stool samples of children with acute respiratory diseases. For phylogenetic analysis, the VP2 genes were sequenced from 69 HBoV-positive NPA samples. The qualitative results of the real-time HBoV PCR were in good agreement with a conventional HBoV PCR. They found that 12% of the NPA were positive for HBoV DNA by both techniques.

Söderlund-Venermo et al., [23] reported that the unique region in VP1 is less immunogenic than the major virus capsid protein VP2, Also he expressed HBoV VP2 viral like particles (VLPs) in insect cells for use in IgM and IgG ELISA that are superior to immunoblots in diagnostic performance.

In the present study 18 (18%) out of the 100 serum samples of the patients with respiratory tract manifestations were positive for HBoV IgM antibodies by ELISA. There was a significant difference between the patient and the control group (p < 0.001). The mean serum concentration of IgM antibodies against HBoV, in the two groups was (0.28 ± 0.06 vs 0.07 ± 0.01). There was also a highly significant association between PCR and ELISA techniques (p < 0.001 and agreement = 96%). Söderlund-Venermo et al., [23] diagnosed acute primary HBoV infections in 49 (19%) of 258 children with expiratory wheezing, and Lindner et al., [24] detected IgM against HBoV by ELISA in 10/24 (42%) of sera samples obtained from HBoV DNA-positive children and infants with infections of the respiratory tract.

In the present study sensitivity, specificity, positive predictive value and efficacy of ELISA were 81.8%,100%,100% and 97.7% respectively Lindner et al., [16] had reported diagnostic sensitivity and specificity of their antibody ELISA were as high as 97% and 99.5%, respectively; positive predictive value was 97% by using ELISA among wheezing children.

## Conclusion

HBoV infections could be diagnosed in NPA by conventional PCR as a rapid and sensitive technique. ELISA was a reliable serologic analysis for diagnosis of acute HBoV infection by estimation IgM antibodies in serum.

## Methods

The present case - control study was conducted on 100 patients aged from 1 to 5 years (61 males and 39 females) with a mean ± SD age of (2.7 ± 1.1) attending the Pediatric outpatient clinic, Ain Shams University Hospitals, Cairo, Egypt in the period from September 2009 to May 2010. Patients were suffering from lower respiratory tract infection together with upper respiratory tract manifestations such as cough, running nose and fever. In addition to 50 apparently healthy children (32 males and 18 females) matched in age with the patients as a control group.

All patients were subjected to the following after their written consent, full history taking and thorough clinical examination, complete blood picture, chest plain X-ray. Nasopharyngeal aspirate (NPA) samples collected from both patient and control groups were subjected to microbiological examination and qualitative polymerase chain reaction (PCR) for detection HBoV. Serum samples were collected from both patient and control groups for detection of HBoV- IgM antibodies by ELISA.

### Sample collection

Nasopharyngeal aspirates were collected from patients and control group according to Svensson et al., [25]. Sterile normal saline solution was instilled in one nostril while occluding the other nostril, using a sterile blunt-tipped disposable syringe. Then the patient was instructed to forcibly exhale through the lavaged side into a sterile specimen cup. The sequence was then repeated in the other side of the nose. NPA specimens were examined microbiologically immediately and part of the specimens was stored in aliquots at -70°C for PCR. Two ml blood samples from both patients and control were collected into vacutainer, centrifuged and serum was separated and stored at - 20°C for HBoV-IgM antibodies by ELISA.

### Microbiological examination

It was done by Gram stained smear for detection of pus cells and microorganisms. Quantitative culture of NPA specimens of patients and control was done. Undiluted and tenfold serial dilutions were inoculated with a calibrated loop on chocolate agar, blood agar and MacConkey agar plates, after over night incubation in appropriate conditions, Quantitative NPA cultures were considered positive when the growth of 10^6 ^(cfu/ml) or more was observed [26].

### Qualitative polymerase chain reaction (PCR)

DNA was extracted from 200 μl of the NPA samples using the High Pure Viral Nucleic Acid Kit (Roche, Mannheim, Germany) according to the instructions of the manufacturer.

Amplification of hBoV DNA was performed using 50 μl elution volume with the NP-1 primers BoV188F and BoV542R described by Allander et al., [1] (table 8) using the HotStarTaq DNA Polymerase (Qiagen, Hilden, Germany). PCR reactions were carried out in a 50 μl volume consisting of 5 μl extracted DNA, 1× Qiagen HotStar buffer, dNTPs at a final concentration of 200 μM each, 200 pmol of each primer, and 1.5 U of Taq Polymerase. The cycling conditions were 50 cycles (94°C 30 s, 53°C 40 s and 1 min at 72°C) after a preheating step of 10 min at 95°C.

**Table 8:** Primer used for the amplification of HBoV

Primer	Sequence (5'-3')	Amplicon size (bp)	Purpose
HBoV188F	GAGCTCTGTAAGTACTATTAC	354	Qualitative PCR

HBoV542R	CTCTGTGTTGACTGAATACAG	354	Qualitative PCR

After amplification, PCR products were visualized by staining with ethidium bromide on agarose gels. A PCR reaction was considered as positive when a band of the expected size (354 base pairs) was visible.

A plasmid containing the PCR product cloned in the vector PCR 2.1-TOPO (Invitrogen) was used as positive control.

### Enzyme linked immunosorbent assay (ELISA)

HBoV-IgM antibodies were determined by a commercially available immunoglobulin M (IgM) enzyme-linked immunosorbent assay supplied by (Dako, Glostrup, Denmark) for the quantitative determination of IgM antibodies to HBoV in serum. Briefly: serum samples diluted 1:200 in phosphate buffer saline (PBS) and 0.05% Tween (PBST) were applied in duplicate into wells of plates coated with goat anti-human IgM for 60 min at room temperature. After being rinsed 5 times with PBST, biotinylated HBoV viral like particles (VLPs) were applied at a concentration of 25 ng/well and incubated for 45 min at 37°C. Bound antigen was visualized by using horseradish peroxidase-conjugated streptavidin at 1:12,000 in PBST plus 0.5% bovine serum albumin for 45 min at 37°C, followed by *o*-phenylenediamine dihydrochloride and H_2_O_2 _for 15 min at 37°C. The reaction was stopped after 10 min with 0.5 M H_2_SO_4_, and the absorbance at 492 nm were recorded. Cut off absorbance for negative and positive IgM ELISA results were 0.136 and 0.167, respectively.

### Statistical analysis

Association between PCR and ELISA for diagnosis of HBoV infection was evaluated using CHI square test. Significance (p < 0.05). Diagnostic validity test including sensitivity, specificity, positive and negative predictive values were calculated considering the PCR a reference method for diagnosis of HBoV infection. The diagnostic power of both ELISA and PCR was estimated using the efficacy rate.

## Competing interests

The author declares that she has no competing interests.

## Authors' contributions

MZ carried out the enzyme linked immunosorbent assay, qualitative polymerase chain reaction, drafted the manuscript, designed the study and performed the statistical analysis. The author read and approved the final manuscript.
